# Patch Testing in Suspected Allergic Contact Dermatitis to Cosmetics

**DOI:** 10.1155/2014/695387

**Published:** 2014-09-09

**Authors:** Pramod Kumar, Rekha Paulose

**Affiliations:** ^1^Dermatology Department, KMC Hospital, Manipal University, Attavar, Mangalore 575 001, India; ^2^Ahalia Hospital, P.O. Box 2419, Abu Dhabi, UAE

## Abstract

*Background*. Increasing use of cosmetics has contributed to a rise in the incidence of allergic contact dermatitis (ACD) to cosmetics. It is estimated that 1–5.4% of the population is sensitized to a cosmetic ingredient. Patch testing helps to confirm the presence of an allergy and to identify the actual allergens which are chemical mixtures of various ingredients. *Objectives*. The aims of this study are to perform patch testing in suspected ACD to cosmetics and to identify the most common allergen and cosmetic product causing dermatitis. *Methods*. Fifty patients with suspected ACD to cosmetics were patch-tested with 38 antigens of the Indian Cosmetic Series and 12 antigens of the Indian Standard Series. *Results*. The majority (58%) of patients belonged to the 21–40 years age group. The presence of ACD to cosmetics was confirmed in 38 (76%) patients. Face creams (20%), hair dyes (14%), and soaps (12%) were the most commonly implicated. The most common allergens identified were gallate mix (40%), cetrimide (28%), and thiomersal (20%). Out of a total of 2531 patches applied, positive reactions were obtained in 3.75%. *Conclusion*. Incidence of ACD to cosmetics was greater in females. Face creams and hair dyes were the most common cosmetic products implicated. The principal allergens were gallate mix, cetrimide, and thiomersal.

## 1. Introduction

“Cosmetics” are preparations for beautifying the complexion, skin, hair, and nails. They are defined as “articles intended to be rubbed, poured, or sprayed on, introduced into, or otherwise applied to the human body or any part thereof for cleansing, beautifying, promoting attractiveness, or altering the appearance without affecting the body's structure or functions” [1].

The strong desire of individuals to improve their appearance using topical applications has resulted in the production of a variety of cosmetics throughout the world. The majority of these substances are synthetic in nature with ingredients capable of causing sensitization of the skin, thus contributing to the increased incidence of cosmetic dermatitis [2].

It is estimated that 1–5.4% of the population is sensitized to a cosmetic or cosmetic ingredient [2–4]. About 80% of reactions occur in patients aged 20–60 years and are seen more frequently in women [2, 4]. An epidemiologic survey in the UK revealed that 23% of women and 18.8% of men experience some sort of adverse reaction to a personal care product over one year [5]. Commonly used cosmetics like soaps, creams, lipsticks, foundations, sunscreens, perfumes, and eye, hair, and nail cosmetics can cause allergic contact dermatitis. The most frequently identified allergens are fragrances and preservatives [2]. Fragrance in various forms is one of the most common causes of allergies. Fragrance is used in almost all skin care products, cosmetics, and also domestic cleaning agents. This has led to a high incidence of fragrance sensitization [6].

## 2. Materials and Methods

Fifty cases with suspected allergic contact dermatitis (ACD) to cosmetics were included in the study. Cases included those with cosmetic dermatitis affecting a site of application or contact with one or more cosmetics and history of precipitation or exacerbation of the dermatitis with cosmetic use. Patients with active dermatitis were excluded from patch testing until the dermatitis subsided. A detailed history regarding symptoms and cutaneous lesions was taken. Information regarding cosmetics used, duration of use, frequency of application, and precipitation or exacerbation of the dermatitis on cosmetic use was noted.

Patch testing was performed in all cases with a total of 50 allergens (Table 1), 38 allergens of the Cosmetic Series and 12 allergens of the Indian Standard Series which are common constituents of cosmetics, as per the recommendations of CODFI (Contact and Occupational Dermatoses Forum of India). The patch test kit was procured by order from Systopic Laboratories Pvt. Ltd., New Delhi. The kit consists of 42 solid allergens and 8 liquid allergens. Wherever indicated, the suspected cosmetic itself was used.

Five patch test units each consisting of ten aluminum chambers mounted on microporous tape were required per patient. The allergens were applied as 5 mm length of solid allergen from the syringes or one full drop of liquid allergen on a filter paper disc on the chamber.

After explaining the procedure in detail to the patient, informed consent was taken. The back of the patient was cleaned with spirit and five patch test units with a total of fifty antigens were applied, four on the upper back and one on the lower back. Wherever possible, a sample of the cosmetic suspected to cause the dermatitis was also included in the patch test.

The patch test units were removed after 48 hours (D2) and readings were taken one hour after removal. The patch test reactions were read according to the recommendations of the International Contact Dermatitis Research Group. The diagnosis of ACD to cosmetics was confirmed based on a positive result.

Photopatch testing and reading at D3 and D7 were not done.

## 3. Results

Age of the patients in the study group ranged from 13 to 67 years. The majority of patients were in the 21–40 years age group (58%). Among males, the majority (31.58%) belonged to the 41–50 years age group while among females the majority (38.71%) belonged to the 21–30 years age group. There were 19 males and 31 females with male : female ratio of 1 : 1.63.

The total duration of dermatitis was less than 1 year in 30 (60%) patients. The minimum duration was 1 month and the maximum duration was 20 years. The mean duration was found to be 23 months.

The most common symptom in both males and females was itching which was present in 17 (89.4%) of males and 28 (90.32%) of females. Other common symptoms were photosensitivity and burning which were present in 21 (42%) and 10 (20%) of the patients, respectively. A higher percentage of males (63.16%) gave history of photosensitivity as compared to females (29.03%). Atopy or asthma was present in 8 (16%) patients, which included 4 males and 4 females.

The face was the most common site of involvement in both males (47.37%) and females (61.29%). Face and neck (21.05%) and scalp (10.53%) were more commonly involved in males, while hands (9.68%) were more commonly involved in females.

The most common lesions in both males and females were erythema and papules. The most common secondary lesions in males were hyperpigmentation (26.32%) and crusting (26.32%) and in females the most common secondary lesions were scaling (41.94%) and hyperpigmentation (29.03%). Secondary infection of the lesions was present in 7 (14%) patients.

Soap was the most common cosmetic used in both males (84.21%) and females (100%). Other cosmetics commonly used in males were hair dyes (52.63%), shaving creams (68.42%), and shampoos (31.58%). Frequently used cosmetics in females were face creams (70.97%), perfumes (41.94%), and shampoos (54.84%). Bulk of males (42.11%) had suspected allergy to hair dye, whereas face creams (45.16%) were the most commonly suspected cosmetics in females. Eighty percent of hair dye users had assumed ACD to hair dye. Incidence of ACD among users of face creams (60%), shaving creams (46.15%), and perfumes (26.32%) was also high.

Allergic contact dermatitis was confirmed in 38 cases (76%) of the study group who had positive patch test reactions, either antigens of the Cosmetic Series or the suspected cosmetic product or both. Overall, 84.21% of males and 70.97% of females had allergy to cosmetics.

In the study group, face creams (20%), hair dyes (14%), and soaps (12%) were the most common cosmetics causing allergic contact dermatitis. Shaving creams (10%), perfumes (8%), and lipsticks (4%) were the other common cosmetics identified.

On performing patch tests with the Standard Cosmetic Series in the study group, the most common antigens giving positive reactions were gallate mix (40%), cetrimide (28%), thiomersal (20%), and paraphenylenediamine (14%). On comparing positive reactions in males and females, gallate mix was the most common allergen in both groups, 47.37% and 35.48%, respectively. Other common allergens in males were cetrimide (31.58%) and paraphenylenediamine (31.58%). Cetrimide (25.81%) and thiomersal (22.58%) were the other common allergens in females.

Thirty-one patients were patch-tested with their personal cosmetics (Table 2). Twenty-two patients (71%) gave a positive reaction, thus confirming the presence of allergic contact dermatitis to cosmetics. On correlating the positive reactions to the suspected cosmetic and the positive reactions to ingredients of cosmetics, a definite causal link was established in many cases. Gallate mix was the most commonly identified allergen in face creams, paraphenylenediamine in hair dyes, gallate mix and cetrimide in shaving creams, and thiomersal in perfumes. Face creams and hair dyes gave maximum number of positive reactions in females and males, respectively.

Among the positive reactions, “1+” reactions were the most common, occurring in 67 (70.53%) patches. “2+” reactions and “3+” reactions were seen in 16 (16.84%) and 12 (12.63%) patches, respectively. Out of a total of 2531 patches applied, positive reactions were obtained in 3.75% (95/2531) patches.

## 4. Discussion

In our study of 50 patients, 19 (38%) were males and 31 (62%) were females. The male : female ratio was 1 : 1.63. Allergic contact dermatitis to cosmetics is more common in females as compared to males [2]; this was also observed in our study where females outnumbered males. In a cross-sectional retrospective study done by the North American Contact Dermatitis Group, the site and category of cosmetics differed somewhat by gender [7].

Age of the patients in the study group ranged from 13 to 67 years. In a study conducted by Adams and Maibach [8], the majority of patients with cosmetic reactions were distributed over the age group 20 to 60 years. In this study, the majority of patients (58%) belonged to the age group of 21–40 years. The majority of males (31.58%) belonged to the 41–50 years age group, whereas the majority of females (38.71%) belonged to the 21–30 years age group. The reason for this could be that the majority of males in the study had allergy to hair dyes which in most cases were first used after the age of 30 years.

Adams and Maibach [8] reported that the duration of the dermatitis prior to consulting the dermatologist was 8 days or longer in nearly all cases. In our study, the duration of dermatitis was less than one year in sixty percent of patients. Itching, which is a manifestation of allergy, was present in 45 (90%) patients of the study group. Other symptoms included photosensitivity (42%), burning (20%), pain (6%), and oozing (4%). A higher percentage of males (63.16%) gave history of photosensitivity as compared to females (29.03%). Photopatch testing was, however, not done in this study as it was out of scope of this protocol. de Groot et al. [9] reported that itching was the most frequent subjective symptom in patients with contact allergy to cosmetics.

The face is the most frequently involved site of cosmetic dermatitis [10]. In this study, face was involved in 56% of cases followed by face along with neck in 10%, face and hands in 8%, and only hands in 6%. Other sites included neck (4%) and scalp (4%). Face was the most common site affected in both males (47.37%) and females (61.29%). Face and neck (21.05%) and scalp (10.53%), the sites for hair dye allergy, were commonly involved in males, whereas hands (9.68%) were exclusively involved in females.

de Groot et al. [9] found that the most frequently reported objective symptom was erythema (61%) followed by scaling (19.3%) and pimples (14.2%). In our study, erythema (52%) was the most common objective symptom followed by papules in 40% and scaling in 34%. Other common primary lesions included plaques (20%), macules (18%), vesicles (10%), and pustules (6%). Secondary lesions commonly seen included hyperpigmentation (28%), crusting (12%), hypopigmentation (10%), and excoriation (10%).

Soap was the most common cosmetic used in both males (84.21%) and females (100%). Other commonly used cosmetics included face creams (50%), shampoos (64%), perfumes (38%), and bindi/sindoor/kumkum (32%). The prevalence of face cream usage was high in the females of the study group (70.97%), whereas hair dye usage was common in males (52.63%).

Mehta and Reddy [11] in their study on the pattern of cosmetic sensitivity in Indian patients reported that bindi, hair dye, and face creams were the most commonly suspected cosmetics in contact dermatitis due to cosmetics. In our study, face creams (30%), hair dyes (16%), and soaps (14%) were the most frequently suspected cosmetics. Males (42.11%) commonly suspected allergy to hair dye whereas females (45.16%) suspected allergy to face cream. The incidence of suspected allergic contact dermatitis was the highest among hair dye users (80%). High incidence was also seen in users of face creams (60%), shaving creams (46.15%), and perfumes (26.32%).

Several studies [10–12] have reported that skin care products (moisturizing and cleansing cream/lotion/milk) account for the majority of cases of contact allergy to cosmetics. This was confirmed in our study where face creams (20%) were the most common cosmetic causing allergic contact dermatitis. Other common cosmetics causing allergy were hair dye (14%), soap (12%), shaving cream (10%), and perfume (8%). Hair dye, shaving cream, and perfume were the common causative agents in males, whereas face cream, soap, perfume, and lipstick were the common causative agents in females.

The most frequently identified cosmetic allergens are fragrances and preservatives [2]. In our study, gallate mix, an antioxidant, was the most common allergen. It was positive in 40% of the study group. This is probably due to the presence of propyl gallate as an antioxidant in skin creams. The other common allergens in cosmetics identified by patch testing included cetrimide (28%), thiomersal (20%), and paraphenylenediamine (14%).

In thirty-one patients, the suspected cosmetics were also included in the patch test. The cosmetics were tested “as is.” Twenty-two patients (71%) gave a positive reaction to the suspected cosmetic, thus confirming the presence of cosmetic contact dermatitis. In a study conducted by Mehta and Reddy [11], patch testing with suspected cosmetics gave positive results in 50% of patients. In four cases, patients had one or more positive reactions to antigens of the cosmetic series but did not react to the suspected cosmetic product. The reason for this probably could be that the concentration of the antigen in the cosmetic was too low to elicit a positive patch test reaction.

An attempt was made to correlate the positive results of the cosmetics tested and the ingredients of cosmetics. A definite causal link was obtained in some cases such as face cream with gallate mix, shaving cream with gallate mix and cetrimide, hair dye with paraphenylenediamine, and perfume with thiomersal. Propyl gallate is an allergen in liposome containing skin creams [13]. The majority of allergy to hair dyes is caused by PPD [13].

In a study on adverse reactions to cosmetics by Dogra et al. [14], out of 2065 patches applied, positive results were obtained in 3.2% patches with standard cosmetic kit and 3.3% patches with various cosmetics. Out of a total number of 2531 patches applied in our study, positive reactions were obtained in 3.75% (95/2531) patches.

A study in Seoul found that not all antigens that are found in cosmetics are actually available in patch test kits and that these could be potential allergens which go undetected; hence, a need for regular modification of testing kits to reflect ingredients in the cosmetics is advocated [15]. In India, a study on pediatric contact dermatitis revealed that children could be sensitive to some of the cosmetic antigens and the sensitization may happen due to early age of cosmetic use [16]. Citrus being a very commonly used substance in fragrance industries should be tested for in all those who have a positive reaction to fragrance mix [17].

## 5. Conclusion

In a short communication on this study, the authors [18] highlighted the necessity for patch testing and careful use of cosmetics in India. Recent studies suggest increased incidence of cosmetic dermatitis and also of newer antigens that cause allergies [19, 20]. Patch testing is an important investigation in patients with suspected allergic contact dermatitis to cosmetics and remains a gold standard [21, 22]. In a growing economy like that of India where the market for cosmetics especially fairness creams and hair cosmetics is in high demand, the reports on cosmetic dermatitis are insignificant [23, 24]. The authors would like to make more detailed analysis and interpretation of their study to emphasize the importance of patch testing in all suspected cases and recommend use of the suspected cosmetic itself for patch testing.

## Figures and Tables

**Table 1:** List of antigens used (1–38 Cosmetic Series, 39–50 Standard Series).

Number	Antigen
1	Control
2	Amercholl
3	Benzyl alcohol
4	Benzyl salicylate
5	Bronopol
6	Butylated hydroxyl anisole
7	Butylated hydroxyl toluene
8	Cetyl alcohol
9	Chloroacetamide
10	Chloroxylenol
11	Gallate mix
12	Geranium oil
13	Oxybenzone
14	Benzotriazole
15	Imidazolidinyl urea
16	Isopropyl myristate
17	Jasmine absolute
18	Lavender absolute
19	Musk mix
20	Phenyl salicylate
21	Polyxyethylenesorbitoal oleate
22	Rose oil
23	Sorbic acid
24	Sorbitan monooleate
25	Sorbitan sesquioleate
26	Stearyl alcohol
27	*tert*-Butylhydroquinone
28	Thiomersal
29	Triclosan
30	Triethanolamine
31	Vanillin
32	Cetrimide
33	Jasmine synthetic
34	Hexamine
35	Diazolidinyl urea
36	Chlorhexidine digluconate
37	Phenyl mercuric acetate
38	Cocamidopropyl betaine
39	Fragrance mix
40	Parabens
41	Propylene glycol
42	PEG-400
43	Chlorocresol
44	Wool alcohol
45	Balsam of Peru
46	Kathon CG
47	Ethylenediamine dihydrochloride
48	Quarternium15
49	Formaldehyde
50	4-Phenylenediamine

**Table 2:** Results of patch testing with patient's own cosmetics.

Suspected cosmetic	Patients tested			Positivity			Suspected antigens
	M	F	Total	M	F	Total	
Face cream	1	12	13	1	7	8	Gallate mix, cetrimide, and thiomersal

Hair dye	7	—	7	5	—	5	Paraphenylenediamine and gallate mix

Shaving cream	5	—	5	5	—	5	Gallate mix and cetrimide

Perfume	—	2	2	—	—	—	Thiomersal and gallate mix

Nail polish	—	1	1	—	1	1	Gallate mix and *tert*-butylhydroquinone

Foundation cream	—	1	1	—	1	1	Gallate mix and cetrimide

Kumkum/bindi	—	2	2	—	1	1	*Paraphenylene*diamine
